# Milestones and Horizons: Highlights from the 4th International Children’s Palliative Care Network Conference in Manila, Philippines

**DOI:** 10.3332/ecancer.2026.2126

**Published:** 2026-05-20

**Authors:** Callie Daniels-Howell, Rumalie Corvera, Lizzie Chambers, Alex Daniels, Rhea Jayma, B-Etta ‘Babes’ Ayon, Nenacia Nirena Ranali ‘Rana’ Mendoza, Fatima Lorenzo, Liza Naranjo, Carmen Auste, Poh Heng Chong, Mike Palfreman, Justin N Baker, Michael J McNeil, Megan Doherty, Julia Downing

**Affiliations:** 1 The International Children’s Palliative Care Network, BS1 2NT Bristol, UK; 2 The Ruth Foundation, Muntinlupa 1771, Philippines; 3 National Children’s Hospital, Metro Manila 1113, Philippines; 4 Kythe Foundation, Quezon City, Philippines; 5 National Palliative and Hospice Care Council (Hospice Philippines), Muntinupa 1780, Philippines; 6 Cancer Warriors Foundation, Manila 4217, Philippines; 7 Asia Pacific Hospice Palliative Care Network, 168583 Singapore; 8 Division of Quality of Life and Pediatric Palliative Care, Stanford School of Medicine and Stanford Medicine Children’s Health, Palo Alto, CA, USA; 9 Division of Quality of Life and Palliative Care, St. Jude Children’s Research Hospital, Memphis, TN, USA; 10 Department of Global Pediatric Medicine, St. Jude Children’s Research Hospital, Memphis, TN 38105, USA; 11 Two Worlds Cancer Collaboration, North Vancouver, Canada; 12 Children’s Hospital of Eastern Ontario, Ottawa, Canada; a https://orcid.org/0000-0003-3238-9375; b https://orcid.org/0009-0005-5872-2051; c https://orcid.org/0009-0006-3840-2760; d https://orcid.org/0000-0003-4241-3295; e https://orcid.org/0000-0002-6584-6483; f https://orcid.org/0000-0001-8817-1995; g https://orcid.org/0000-0003-3905-2169; h https://orcid.org/0000-0002-3450-785X

**Keywords:** palliative care, children, international, collaboration, Philippines, research, innovation

## Abstract

The International Children’s Palliative Care Network (ICPCN) held its 4th international conference in Manila, Philippines (12th–15th November 2025), in partnership with The Ruth Foundation. The conference, ‘Milestones & Horizons,’ marked ICPCN’s 20th anniversary and convened 419 delegates from 49 countries across all World Health Organisation regions to celebrate the progress of children’s palliative care (CPC), while identifying priorities for equitable access for the 21 million children with life-threatening and life-limiting conditions. Hosted in the Philippines, it showcased local developments, including the National Children’s Hospital Pediatric Palliative Care Unit and community models, catalysing CPC’s formal recognition as a specialty by the Philippine Pediatric Society. The conference featured 155 accepted abstracts (63 oral, 26 rapid-fire and 46 posters), 10 keynotes, 5 plenary panels, 7 ‘Meet the Expert’ sessions and 9 workshops on leadership, research, advocacy, spiritual care, touch therapy and equity. Sessions highlighted milestones in system building, service integration, interdisciplinary collaboration and family-informed research, alongside horizons in digital innovation, humanitarian reach and children’s voices. Several sessions highlighted significant CPC ‘Milestones’, focusing on health system strengthening, service integration and diverse multidisciplinary research. Around the theme of ‘Horizons’, key conference sessions explored future directions such as digital innovation, care in humanitarian settings, interprofessional leadership and amplifying children’s voices through co-design and creative therapies. Conference evaluations emphasised the high value of the in-person event with the opportunity for increasing personal motivation, peer support and new collaborations. The 2025 ICPCN conference demonstrated CPC’s maturation through interdisciplinary collaboration while catalysing local progress in the Philippines. Critically, the conference demonstrated the irreplaceable value of in-person convening for relationship-building across diverse organisations, creating momentum toward shared goals of universal access and health system integration. Evaluations confirmed this collaborative energy as uniquely motivating, fostering cross-border partnerships and actionable commitments amid constrained resources, climate challenges and humanitarian needs.

## Introduction

The International Children’s Palliative Care Network (ICPCN) held its 4th international conference in Manila, Philippines, from 12th–15th November 2025, in partnership with The Ruth Foundation (TRF) for Palliative and Hospice Care. The meeting marked ICPCN’s 20th anniversary and was organised around the theme ‘Milestones & Horizons,’ inviting delegates to reflect on how far children’s palliative care (CPC) has come and what remains to be done to ensure that all 21 million children worldwide living with life-limiting or life-threatening conditions can access quality palliative care [1–3]. The choice of the Philippines, a lower-middle income country with an evolving CPC landscape, as host location and of TRF as partner, embodied this dual focus on celebrating achievements and confronting current and future gaps.

The 2014 World Health Assembly Resolution on palliative care, calls on Member States to integrate palliative care as a core component of health systems to achieve universal health coverage, with particular attention to primary and community-based care [4–6]. This conference signals a renewed focus on identifying, sharing and building upon key innovations in CPC, focusing on integrated service delivery, policy, education and research, while recognising the continued need to expand palliative care access in low-resource and humanitarian settings. Against this backdrop, the ‘Milestones & Horizons’ theme offered a unifying lens through which plenaries, workshops and free papers could situate local experiences within a shared global trajectory.

## Key messages: Milestones and Horizons

### Milestones: 20 years of global progress

The conference highlighted substantial global progress in CPC, particularly in low- and middle-income countries over ICPCN’s 20 years of convening interdisciplinary collaboration. System building through national standards, training and community programmes has scaled services from pilots to sustainable frameworks. Service innovations embedding CPC within hospitals, hospices and home care have improved accessibility and quality of care through creative, family-centred models. Research milestones incorporating family perspectives through validated outcome measures have built a globally representative evidence base, shifting from need description to policy-shaping practice. Palliative care has been recognised as an essential component of Universal Health Coverage and a key consideration in the care of children with serious illnesses. With growing multidisciplinary CPC teams worldwide and routine integration into global disease initiatives, CPC is successfully becoming embedded in standard care.

### Horizons: leadership and collaboration will be transformational

Despite 20 years of progress, more than 90% of children with serious illnesses are still not able to access palliative care, mainly concentrated in resource-limited settings [7]. For truly transformative global change, making palliative care accessible to all children everywhere, leadership and collaboration are key. Training, empowering and supporting the next generation of palliative care leaders through regional workshops remains essential for sustainable service growth, improved access to essential medicines and care integration across health systems adapting to constrained resources and humanitarian challenges. The ICPCN 2025 conference contributed directly to these horizons through capacity-building workshops on advocacy, leadership, education and research, while intentionally highlighting examples of collaboration and creating opportunities for new interdisciplinary partnerships. Collaboration, connecting health, social care, education, governments, academics and families, is vital, sharing innovations to accelerate capacity while ensuring equitable reach to marginalised children through interdisciplinary, family-informed approaches. Amid constrained resources, climate crisis and humanitarian emergencies, horizons demand coordinated collaboration across digital innovation for remote access, humanitarian care continuity, education, research capacity through shared evaluation tools and sustained family empowerment.

## The International Children’s Palliative Care Network

The ICPCN was founded in 2005 as the global action network dedicated specifically to CPC. Its mandate is to promote the development, integration and recognition of CPC worldwide through communication, advocacy, research, education, strategic development and collaboration. ICPCN is widely acknowledged as the leading global voice for CPC [8, 9]. Its vision is a world in which every child and young person with a life-limiting or life-threatening condition and their family, can access high-quality, holistic palliative care, addressing physical, emotional, spiritual and developmental needs, regardless of geography or diagnosis.

Over the past 20 years, ICPCN has grown into a broad, multidisciplinary network with 540 organisational and 6,200 individual members in more than 120 countries, linking individuals, specialist CPC teams, generalist providers, academic institutions, non-governmental organisations (NGOs) and parent advocates [10]. Through its online education programme, regional trainings and contributions to foundational resources such as CPC: An International Case-Based Manual, ICPCN has supported thousands of practitioners to build skills and services in varied contexts [11–13]. Its research and mapping activities have documented where CPC exists, where gaps persist and what families say they need, while advocacy efforts have contributed to global policy recognition of CPC and to national initiatives in multiple regions [4, 7].

International conferences are a key mechanism for ICPCN to enact its mission. They create protected space for clinicians, researchers, educators, advocates, policymakers and family representatives to share innovations, interrogate challenges and co-design future priorities. The 2025 ICPCN Manila conference, following previous meetings in India, Argentina and South Africa, was intentionally structured to showcase work from all World Health Organisation (WHO) regions, with particular emphasis on low and middle-income settings, multidisciplinary frameworks and family- and child-led initiatives [14–16]. In this way, the conference itself became both a milestone in ICPCN’s organisational history and a platform for articulating new horizons for the field.

## Children’s Palliative Care in the Philippines

The Philippines is a lower-middle income country in Southeast Asia with an estimated population of approximately 114 million in 2025, of whom roughly 30% are under 15 years of age [17, 18]. Muntinlupa, a highly urbanised city in Metro Manila and the site of the 2025 ICPCN conference, had a population of 543,445 at the 2020 census [18]. Childhood cancer incidence is broadly similar to global estimates, at roughly 10–11 new cases per 100,000 children per year, yielding several thousand new cases annually [19, 20]. A recent population-based study of Filipino paediatric patients (2006–2017) reported 5-year overall survival substantially lower than the 75%–90% observed in many high-income countries [21]. These disparities, driven by limited specialist oncologists and referral centres, geographic challenges and financial scarcity, contribute to a high burden of health-related suffering and underpin a substantial unmet need for CPC [19, 22]. Palliative care for all patients and particularly children, remains limited in the Philippines due to few trained specialists, substantial barriers to accessing healthcare and limited availability of opioids [22, 23].

The National Children’s Hospital (NCH) in Quezon City is a government tertiary and teaching hospital that serves as a major public referral centre for paediatric oncology within the national childhood cancer network [24]. It manages several hundred new paediatric cancer cases annually across a wide catchment area [20]. The NCH Paediatric Palliative Care Unit, established in 2020, is reported to be the first dedicated multidisciplinary CPC team in a tertiary government paediatric hospital in the Philippines, providing outpatient consultations, inpatient referral support, symptom management and end-of-life care. In 2025, it began developing a structured home-care programme to strengthen continuity between hospital, home and community [25, 26]. Other tertiary facilities, including the Philippine General Hospital and regional cancer centres, provide elements of paediatric palliative care, often through oncology, pain or general paediatrics services. However, fully resourced multidisciplinary CPC teams remain few and concentrated in urban hubs [20, 24, 27].

TRF for Palliative and Hospice Care, based in Muntinlupa City, is a NGO that has become an important provider and catalyst for palliative care [28]. Established in 2012–2013, TRF is licensed as a social welfare and healthcare provider and focuses on community-based palliative and hospice care for people with life-limiting illness, delivered by multidisciplinary home-visiting teams, alongside education, training and advocacy [28]. Its work is structured around direct care encounters in patients’ homes and community empowerment activities that support other organisations and local providers to develop palliative care services [28, 29].

At the policy level, the National Integrated Cancer Control Act and the Universal Health Care Act, both enacted in 2019, mandate comprehensive cancer control, explicitly including palliative and survivorship care, within the benefits of the national health insurance system [22, 30, 31]. The WHO Regional Office for the Western Pacific is held in Manila, strategically positioning the country to influence regional policy and capacity building across diverse healthcare systems. The Philippines is also a focus country in the WHO Global Initiative for Childhood Cancer, committing to improve survival and reduce suffering through strengthened early diagnosis, treatment and palliative care [32]. However, significant inequities remain in access to specialised CPC across regions [19, 23]. Within this evolving landscape, hospital-based services at centres such as the NCH Pediatric Palliative Care Unit and community programmes led by organisations like TRF represent key, though still partial, responses to the ongoing need for CPC among children in the Philippines. Hosting ICPCN 2025 in Manila amplified this momentum: dignitaries, including the Mayor of Muntinlupa City, Ministry of Education representatives and Philippine Pediatric Society leadership attended, catalysing formal recognition of CPC as a specialty during the conference – marking a national milestone facilitated by ICPCN’s global platform.

## Conference Summary

**Figure 1.**
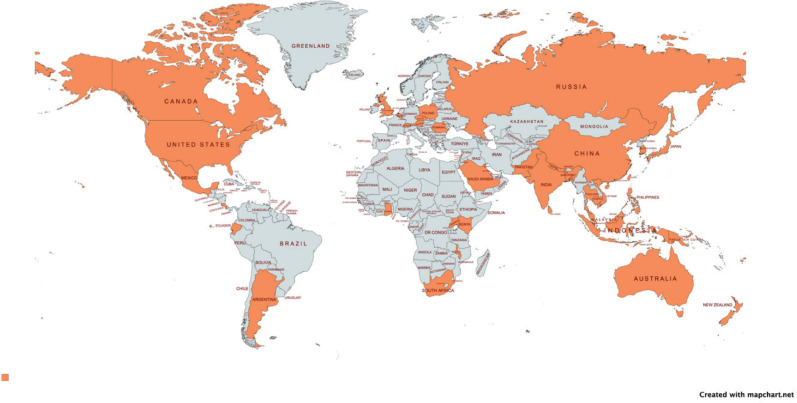
Map of conference delegates’ country representation. Countries represented at the conference were: Argentina, Australia, Austria, Bangladesh, Belgium, Cambodia, Canada, China, Cost Rica, Czechia, Ecuador, Fiji, Ghana, Guernsey, Hong Kong, India, Indonesia, Japan, Kenya, Republic of Korea, Kuwait, Lebanon, Luxembourg, Malawi, Malaysia, Mexico, Nepal, New Zealand, Pakistan, Papua New Guinea, Philippines, Poland, Qatar, Romania, Russia, Saudi Arabia, Singapore, South Africa, Sri Lanka, Switzerland, Taiwan, Thailand, Timor-Leste, Tonga, Uganda, United Kingdom, United States of America, Vanuatu and Vietnam.

The conference attracted 419 delegates from 49 countries across all six WHO regions (Figure 1), representing physicians, nurses, social workers, psychologists, child life specialists, spiritual care providers, advocates, policymakers and educators with varying levels of CPC experience.

The scientific programme reflected both breadth and depth. In total, 155 scientific abstracts were accepted, yielding 63 oral presentations, 26 rapid-fire oral/poster presentations and 46 displayed posters across parallel sessions. These were complemented by ten invited keynote talks, five themed plenary panels and seven ‘Meet the Expert’ roundtable discussions, with speakers from across all six WHO regions, including the Philippines, wider Asia–Pacific, Africa, Europe, Eastern Mediterranean and the Americas [3]. This mixture of formats allowed delegates across levels of experience, geographic regions and disciplines to share work side by side and ensured strong representation from low and middle-income as well as high-income settings.

Among these participants, 225 individuals from 35 countries, representing a 53% response rate, shared reflections through a conference evaluation that vividly captured the meeting’s impact. Delegates described how the scientific programme’s breadth truly deepened their grasp of global CPC trends and innovations, which emerged as the top-ranked learning outcome with over 80% rating it ‘strongly’ or ‘very’ impactful. Doctors and nurses reported the highest gains across professional roles, while attendees with mixed experience levels, from novices to veterans spanning 15+ years, found equal value in understanding how CPC evolves across contexts.

Preconference and in-conference workshops were a defining feature of this meeting and signalled strong investment in capacity-building. Five full- and half-day preconference workshops covered: a regional CPC leadership workshop for Asia [33]; an introductory CPC skills course for clinicians in the Asia-Pacific region; an intensive workshop on integrating touch therapy and paediatric massage into palliative care practice; a ‘Death and grief in schools’ workshop for educators and clinicians and a session on trauma-informed spiritual care for children and families [34]. These drew consistently high attendance across disciplines, with lively small group work and case discussions. Four in-conference workshops then focused on supporting global research, developing advocacy skills, equitable access to CPC training and service-level data tools. The level of engagement in these sessions, including participants bringing real world dilemmas and draft projects for group input, illustrated how in-person convenings can accelerate learning, peer support and concrete action planning beyond what is possible in virtual formats alone.

During the conference, awards were given for the oral presentation and rapid-fire poster presentation that received the highest scores from the panel of reviewers. The best oral presentation abstract was awarded to ‘Whare Kaiao: A New Zealand Indigenous informed paediatric palliative care framework’ [34]. The best rapid-fire poster abstract was awarded to ‘Rhythms of Resilience: A Study on the Impact of Music-based Psychosocial Support offering in Paediatric Palliative Care, Chennai, India’ [34].

On each of the 2 days that posters were displayed, delegates were able to vote for the best poster. These were awarded to ‘The Boy Who Screams ‘NO’ - How Play Therapy Helps to Bridge The Gap of Understanding Between A Dying Child and Care Team’ from Malaysia and to ‘Knowledge, Attitude and Practices Towards Palliative Care in a Tertiary Government Paediatric Hospital in Quezon City, Philippines’ [34].

These awarded abstracts exemplify the conference theme, not only highlighting milestones in CPC development, practice and research but also urging the field forward. These abstracts from Malaysia and India demonstrated the power of creative and arts-based therapies, while work from New Zealand illustrated the importance of incorporating diverse ways of knowing into our care, an opportunity for CPC care providers in diverse contexts globally [34].

### Milestones

Plenary, oral, rapid-fire and poster sessions throughout the conference highlighted the significant, far-reaching milestones in the development of CPC. Presentations showcased progress in global system building, service integration and model innovation and research methods and prominence.

The opening panel of the conference, Milestones and Horizons, highlighted progress and persisting gaps in CPC: growth in services and training, but continuing inequities in access, pain control and support in humanitarian contexts [34]. This laid the foundation for the remainder of the conference, which consolidated and gave the field a sense of shared recognition of global progress.

#### Global system building

Global system building emerged as one of the clearest ‘milestones’ of the ICPCN 2025 conference, showing that CPC can be woven into health systems across very different contexts. Presenters from China, Ghana and Vietnam demonstrated how advocacy, partnerships and training are moving services from small pilots towards recognised programmes, standards and national strategies.

In China, hospice work that began in orphanages has grown into dedicated CPC wards at Hunan Children’s Hospital, a nationally recognised training base, the first home-care centre and a national hospice standard for orphanages, providing a replicable framework for other resource-constrained settings [34]. In Ghana, the #ChilPalCareGhana project used a needs assessment, a community of practice, training, mentorship and clinical placements to catalyse CPC in 57 sites and reach over 12,000 children and families, with the programme extended to consolidate these gains [34, 35]. In Vietnam, a hospital-based CPC consultation service at City Children’s Hospital, drawing referrals from 14 departments, illustrated how a crosscutting team can provide psychosocial support, targeted pain management and culturally congruent home-based end-of-life care within a large tertiary centre [34]. Together with regional leadership initiatives and global collaborations highlighted in plenaries, these examples portray system building as a layered, long-term endeavour rather than a single project.

#### Service integration and model innovation

Within these global systems, service integration and model innovation also featured prominently as a milestone of the field. This illustrates how CPC is being embedded into existing services and reimagined to fit local and evolving realities, such as Costa Rica’s interdisciplinary pain management programme incorporating families, communities and specialist CPC teams to care for children at home, in the community and in hospital. In Kuwait, an interdisciplinary hospice model informed by systematic family feedback and designated family support coordinators working alongside clinical specialists achieved consistently high satisfaction across clinical, psychosocial, spiritual and environmental domains, offering a practical template for family-centred inpatient care [34]. Singapore contributed two notable innovations: a cross-trained therapist delivering integrated music and occupational therapy in the home, which reduced fragmentation while enriching children’s engagement and the Little Flower Care Suite, a paediatric respite unit located within an adult nursing home that showed how thoughtful redesign of existing infrastructure can create much needed inpatient respite [34].

Other abstracts and plenaries highlighted integration within tertiary hospitals and homecare programmes in South Africa, Vietnam and the Philippines, where CPC teams work alongside oncology, intensive care and chronic disease services to provide coordinated symptom management, psychosocial support and transition to homebased care [34]. Linked with sessions on pain, symptom innovation and psychosocial support, these examples suggest a horizon in which specialist CPC is less a standalone niche and more a set of adaptable, team-based models woven through hospitals, hospices and communities.

#### Rigorous evidence base for children’s palliative care

A key milestone visible across the programme was the consolidation of a more rigorous, diverse and globally representative research base in CPC, through which family voices are increasingly shaping evidence and practice. Studies spanned systematic reviews, multicentre quantitative work, mixed methods evaluations and in-depth qualitative inquiries, covering topics from pain knowledge and attitudes to music-based psychosocial interventions, end-of-life quality improvement and social determinants of health [34]. Large-scale initiatives, including St. Jude Global’s Assessing Doctors’ Attitudes on Palliative Treatment study conducted across 127 countries and King’s College London’s Global Health and Palliative Care Research Group, demonstrated exciting milestones in the scale, impact and funding for CPC evidence [34, 36–38]. Tools presented, such as the Children’s Palliative Outcome Scale (C-POS), illustrated how structured assessment, benchmarking and data-driven action planning can be implemented across multiple institutions and countries, strengthening the methodological spine of quality improvement and service design [39, 40]. Systematic and scoping reviews synthesised evidence on bereavement, memory-making and service models, while mixed methods and longitudinal designs evaluated complex interventions in homecare, education and psychosocial support [34]. Importantly, this work came from every WHO region, including low and middle-income and humanitarian settings, signalling a move away from a predominantly high-income evidence base towards a more globally grounded science of CPC. Together, these developments mark a shift from describing needs to testing models, measuring outcomes and building transferable frameworks for practice.

### Horizons

The conference also illustrated and challenged innovation around the future horizons of CPC. These included digital and technological innovation, issues of equity, inclusion and humanitarian reach, leadership, collaboration and global capacity, and the centrality of children’s voices and family empowerment to our work going forward.

#### Digital and technological innovation

Digital and technological innovation emerged as a clear horizon: an area of both promise and ongoing need. Presentations on codesigned adolescent symptom apps, assistive technologies, telehealth platforms, virtual reality for pain and 24/7 nursing phone lines showed how technology can extend reach, personalise communication and mitigate distance, especially for families far from specialist centres [34]. The EU-funded PALLIAKID consortium exemplifies this frontier, developing predictive models for care needs, family engagement platforms and cross-border data standards that integrate digital tools for harmonised outcome measurement [34]. A codesigned adolescent symptom and communication app, ‘Pain in the App,’ demonstrated how humour, flexible symptom logging and user-controlled data sharing can make it easier for young people to express distress and engage with their teams [34]. Telehealth emerged as a durable model beyond the pandemic, with multicentre studies confirming its acceptability for complex CPC follow-up and 24-hour nursing phone lines and virtual reality interventions offering new ways to bridge distance and manage symptoms [34].

Alongside hybrid fellowships and online education initiatives, these examples suggest a future in which digital platforms, remote expertise and assistive technologies are routine elements of CPC. Yet across sessions, speakers emphasised that digital tools are not yet routinely embedded in CPC and that significant work remains to ensure equitable access, robust evaluation and integration into workflows and funding models. Priorities identified included codesign with children and families, addressing connectivity and device gaps, building staff confidence in using new tools and generating comparative data on effectiveness and cost. Technology was presented not as an optional addon but as a necessary frontier if CPC is to reach all 21 million children in need, particularly those in remote, fragile or resource-limited settings.

#### Equity, inclusion and humanitarian reach

Equity, inclusion and humanitarian reach were repeatedly named as priority areas of focus for the field [11, 41]. Case studies from Lebanon, Uganda and other fragile settings illustrated how home-based programmes, telehealth, local medicine production and creative medication distribution systems can sustain CPC through economic collapse, pandemics and conflict, while keeping families at the centre [34]. These also exposed profound gaps in access to medicines, trained teams and sustainable funding. Work with adolescents living with HIV in South Africa and with children facing stigma, poverty and violence underscored that social determinants and health‑system barriers remain powerful drivers of suffering, requiring CPC to engage beyond the bedside [34]. Abstracts on rare diseases and medical complexity highlighted disparities between diagnoses and between countries, with children outside oncology or major urban centres often receiving little or no specialised support [34]. Plenaries on culture, faith and humanitarian CPC, together with indigenous-informed education and frameworks from New Zealand, reinforced that equity is not only about geography but also about cultural safety and meaningful partnership with marginalised communities in service design and delivery [34]. The horizon here is ambitious: CPC integrated into universal health coverage, emergency and humanitarian response, with functioning CPC teams and access to basic paediatric formulations in every country, and no child excluded because of diagnosis, geography, status or culture.

#### Leadership, collaboration and global capacity

Leadership and collaboration were framed as essential horizons if the gains of the last 20 years are to translate into universal access. Cross‑regional initiatives such as Bridge Poland–Colombia, South Asia leadership workshops, hybrid fellowships and national standard‑setting efforts in countries like India illustrated how intentional mentoring, shared curricula and twinning relationships can accelerate system development [34]. Global quality projects such as ‘Pediatric Oncology Tool for End-of-life Care Treatment’ and ‘Healing through End-of-Life Care. and Assistance for the Loss of a Child,’ the C-POS along with ICPCN’s mapping, research hub and advocacy work, pointed towards an emerging shared infrastructure for learning and accountability [7, 34, 40]. Presentations on national standards in India, strategic philanthropy from foundations and evolving funding landscapes underscored that sustainable system change depends on aligning technical expertise, advocacy and financial support [34].

However, discussions consistently returned to unmet needs: limited protected time and funding for CPC leadership roles and rigorous research programmes, fragile reliance on a few champions and scarce south–south collaboration compared with north–south partnerships. Priorities for the coming decade included building interprofessional leadership pipelines, embedding CPC in national training and policy frameworks and aligning strategic philanthropy with locally defined agendas rather than short‑term projects. Leadership and collaboration were cast not as optional extras but as preconditions for reaching the horizon of CPC as a routine, sustainable component of child health systems worldwide.

#### Interdisciplinary research visibility and impact

Research emerged as a central horizon for CPC, with sessions highlighting the urgent need for interdisciplinary approaches to generate equitable, actionable evidence globally. Plenaries and workshops highlighted persisting global gaps in research capacity, infrastructure and protected funding, emphasising the need for shared data, mentorship networks and family-led priority setting. Panels such as ‘Addressing Gaps to Measure Suffering’ and ‘Measuring Outcomes and Quality in CPC’ called for harmonised, culturally appropriate outcome measures and wider adoption of service‑embedded evaluation. Interactive sessions including ICPCN’s human-centred design workshop, ‘Co‑creating a Global Research Agenda,’ demonstrated how collaboration across disciplines and income settings can translate evidence into real‑world improvement [34]. Delegates agreed that building sustainable research ecosystems supported by global mentorship, interdisciplinary partnerships and child and family-inclusive methods will be crucial for the next decade. The conference was an opportunity to learn from existing research consortia, including PALLIAKID, St. Jude Global Palliative Care, the C-POS and Australia’s Centre for Research Excellence in Paediatric Palliative Care [34]. These consortia demonstrate what is possible and necessary for the future of CPC research: interdisciplinary collaboration, policy-aligned agendas and innovative family-centred and mixed method implementation science that generates knowledge and expands access to quality CPC. Research is both a horizon and a shared responsibility: a pathway to ensure that every study strengthens care for children, wherever they live.

#### Children’s voices and family empowerment

Amplifying children’s voices and strengthening family empowerment emerged as a defining horizon and a test of whether CPC is truly person‑centred. ICPCN’s book of family stories, Heart Strings, was launched at the conference [42]. The book captures reflections from families and the services that support them in each WHO region, illuminating the impact of hearing from and sharing the diverse ways that CPC provides support in different contexts. Studies using the Life Reflection Card Game with adolescents with Duchenne muscular dystrophy, co‑designed adolescent apps and narrative and participatory research with families demonstrated the transformative potential of tools that enable children and parents to articulate their values, preferences and experiences in their own words and images [34]. Presentations on healthcare clowning, play, music and arts‑based interventions showed how creative modalities uphold identity, joy and connection, even near the end of life, while work on bereavement support in India, Pakistan and remote Australia highlighted the need for proactive, culturally attuned follow‑up for families after a child’s death [34]. Across ethics and decision‑making sessions, speakers stressed that truly family‑centred care requires time, shared responsibility and communication structures that honour relational decision‑making rather than seeing it as a barrier. The horizon identified was clear: a future where children and families routinely co‑design services, tools and research; where their stories drive priorities; and where empowerment is measured not only in satisfaction, but in agency, preparedness and the ability to live, and grieve, with dignity.

## Discussion

The ICPCN 2025 conference portrayed a field that has achieved substantial milestones while remaining clear‑eyed about the horizons still ahead, and highlighted ICPCN’s role in convening, supporting and sustaining this global community. Across plenaries, workshops and free papers, collaboration emerged as the foundational thread: past gains in policy, service development, research and education have all been built through partnerships between hospitals, community organisations, universities, policymakers and national associations, funders and, crucially, children and families themselves. Presentations showed how interdisciplinary, holistic care, attending to physical, psychosocial, spiritual and practical needs of the whole child and family, has deepened over time, becoming more diverse, inclusive and contextually attuned through creative arts, spiritual care, play, bereavement support and community‑based models across regions and income settings. Cross‑cutting themes of cultural and contextual adaptation, methodological innovation, and sustained capacity‑building and systems-level change underscored a shared recognition that CPC must be both evidence-based and locally grounded if it is to be equitable and effective.

Conference evaluation responses reinforced the value of convening in person. Delegates from a wide range of countries, professional roles and experience levels described how hearing concrete examples from other settings provided rare and powerful learning that is otherwise difficult to access, especially in countries where CPC remains nascent or fragile. Attendees highlighted the motivational impact of seeing what is possible and the sense of belonging that comes from being reminded they are part of a global community working towards shared goals, rather than isolated individuals or teams struggling alone. Many reflected that the relationships, informal conversations and joint problem‑solving that happened between sessions were as important as the formal content for strengthening a sense of teamwork and collaboration across borders. As one delegate said, ‘It was inspiring to connect with professionals from around the world who share the same goal, mission, and vision – to uplift and advocate for CPC. Hearing diverse perspectives, learning best practices, and witnessing a unified commitment to improving the quality of life of palliative children truly reaffirmed the importance of our work.’

## Conclusion

The conclusion of the conference brought these threads together. The field of CPC has come far since its beginnings and over ICPCN’s 20 years as a coordinating body, yet, as the ‘Milestones & Horizons’ theme underscored, there is still far to go. As a member of the scientific committee reflected, ‘horizons are the commitments we are now brave enough to say out loud’: universal access to pain relief and holistic care, integration into universal health coverage and humanitarian response, meaningful inclusion of marginalised communities and protection and support for the workforce that carries this work. Achieving these goals, articulated against the backdrop of the World Health Assembly Resolution and ICPCN’s two decades of work, cannot be the task of any single programme or country. Conferences like this one not only facilitate learning and innovation for individual practices and services; they remind participants that they are part of a grand, fierce, deeply compassionate community of people around the world who want to see a world where children can live fully, even in the face of serious illness.

In the closing remarks, collaboration was a key message, emphasising that in low‑ and middle‑income countries ‘collaboration is not optional, but the very path that makes palliative care possible,’ a truth that increasingly applies across all settings in an era of constrained resources, climate crisis and humanitarian emergencies. The conference echoed the reminder that children and their families are the first and most important collaborators in CPC; their perspectives and priorities must shape services, research and advocacy if future horizons are to be meaningful. As was observed, ‘creativity emerges when you refuse to let suffering be the end of the story’ – a fitting summary of both the milestones celebrated in Manila and the horizons the global CPC community now commits itself to pursue together.

## List of abbreviations

CPC, Children’s palliative care; ICPCN, International Children’s Palliative Care Network; NCH, National Children’s Hospital of the Philippines; TRF, The Ruth Foundation; WHO, World Health Organisation.

## Conflicts of interest

There are no conflicts of interest to declare.

## Funding

No funding was received for the preparation and publication of this report.
